# USING RESEARCH TO ADVANCE THE DEMENTIA-FRIENDLY COMMUNITY MOVEMENT AT THE LOCAL, NATIONAL, AND INTERNATIONAL LEVEL

**DOI:** 10.1093/geroni/igac059.087

**Published:** 2022-12-20

**Authors:** Clara Scher, Emily Greenfield, Joseph Gaugler

## Abstract

It is estimated that more than 55 million people worldwide are living with dementia. To address the social and health needs of individuals living with dementia and their care partners, researchers, policymakers and advocates have championed dementia-friendly communities (DFCs) as a population-level response. DFCs promote the well-being of those living with dementia, empower all members of the community to celebrate the capabilities of persons with dementia, and encourage individuals living with dementia to engage in their communities. The objective of this symposium is to describe the ways in which research can help to advance the dementia-friendly movement at the local, national, and international levels. First, Scher & Greenfield will describe the dimensions of implementation of DFCs in Massachusetts with implications for program monitoring and process evaluation. Second, Epps & colleagues will discuss the process of developing a person-centered tool to evaluate the impact of dementia-friendly programs in faith-based communities. Third, Somerville & colleagues will present findings from a study of community and organizational factors related to dementia-friendly readiness in community-based senior centers. Finally, Sun & colleagues will discuss the barriers and facilitators to implementation of DFCs in the USA during the COVID-19 pandemic. Taken together, these studies demonstrate the utility of quantitative and qualitative research methodologies to elucidate how and to what extent DFCs are implemented. Findings have implications for examining the population health impact of DFC efforts, as well as for attending to issues of health disparities and aging equity in the uptake, implementation, and sustainability of DFC initiatives.

